# YAP is essential for TGF‐β‐induced retinal fibrosis in diabetic rats via promoting the fibrogenic activity of Müller cells

**DOI:** 10.1111/jcmm.15739

**Published:** 2020-09-20

**Authors:** Wei Zhang, Yichun Kong

**Affiliations:** ^1^ Tianjin Eye Hospital Tianjin Key Lab of Ophthalmology and Visual Science Tianjin Eye Institute Clinical College of Ophthalmology Tianjin Medical University Tianjin China; ^2^ Tianjin NanKai Hospital NanKai Hospital Tianjin Medical University Nankai university affiliated Nankai hospital Tianjin China

**Keywords:** fibrosis, retina, retinal Müller cells, transforming growth factor‐β, Yes‐associated protein

## Abstract

The purpose of this study was to investigate whether Yes‐associated protein (YAP) activation and proliferation of retinal Müller cells play a role in the development of TGF‐β‐induced retinal fibrosis. We studied the effects of YAP activation on retinal fibrosis in diabetic rats and human retinal Müller cells (hMCs) in vitro. The retinal expression of YAP and fibrogenic molecules in rats was detected using Western blotting and immunohistochemistry. After treatment with transforming growth factor‐β1 (TGF‐β1), the levels of fibrogenic molecules, and the activation of YAP and PI3K/Akt signalling pathway in hMCs were detected with Western blotting. The effect of YAP on retinal fibrotic changes was evaluated using YAP knockdown experiments and YAP inhibitors. Results showed that YAP expression was increased in the retina of diabetic rats along with increased retinal fibrosis. In cultured hMCs, YAP inhibition suppressed TGF‐β1‐stimulated hMC differentiation to myofibroblasts and extracellular matrix (ECM) production, while YAP activation promoted hMC differentiation and ECM production independent of TGF‐β1. Furthermore, hMCs cultured on a gel with greater stiffness differentiated into myofibroblasts in a YAP‐dependent manner. In diabetic rats, treatment with the YAP inhibitor verteporfin suppressed retinal fibrogenesis. In addition, the TGF‐β1‐induced PI3K/Akt signalling pathway mediated YAP activation as well as expression of fibrogenic molecules. The interaction between ECM stiffness and YAP forms a feed‐forward process leading to retinal fibrosis. Our work highlights YAP as an essential regulator of pro‐fibrotic responses in TGF‐β‐induced retinal fibrosis.

## 1 INTRODUCTION

Diabetic retinopathy (DR) can lead to blindness through intraocular angiogenesis, macular oedema and scar formation. Ischaemia‐induced pathological neovascularization and extracellular matrix (ECM) production are accompanied by the formation of the epiretinal vascular membrane within the vitreo‐retinal interface, which is a pathological feature of proliferative DR (PDR). This often results in severe vision loss because of tractional retinal detachment or vitreous haemorrhage.^1^ PDR appears to be a wound healing response in which new blood vessel formation is associated with the influx of myofibroblasts and inflammatory cells into the retina.^2^ The resultant epiretinal fibrocellular membrane consists of a rigid and contracted scar tissue with contracted myofibroblasts and excessive ECM deposition.^3^ This membrane may damage the retinal structure, leading to photoreceptor destruction and retinal fold formation, eventually causing diabetic tractional retinal detachment.

Retinal Müller cells (MCs) play a key role in retinal fibrosis due to their involvement in maintaining retinal homeostasis.^4^ MCs have been found to assume the role of fibroblasts, which exist in the retina.^5^ In particular, MCs have been reported to have the ability to transform into myofibroblast‐like cell types, characterized by the contraction of the epiretinal membrane, due to the expression of α‐smooth muscle actin (α‐SMA).^6^ Myofibroblasts play an important role in wound healing and fibrosis, which fully clarifies the function of ECM protein secretion and resulting tissue contraction. The presence and formation of myofibroblasts are driven by a number of growth factors and pro‐fibrotic cytokines, such as basic fibroblast growth factor, platelet‐derived growth factor (PDGF) and transforming growth factor‐β (TGF‐β).^7^ TGF‐β has been shown to be the most important activator of myofibroblast formation.^8^ Most fibrous retinopathies are related to TGF‐β‐induced formation of myofibroblasts and retinal elastic modulus, suggesting that an increase in tissue stiffness may promote retinal fibrosis.^9^ However, it is still unclear how tissue stiffening in the retina activates MCs. Furthermore, the origin of sensors that recognize ECM formation to induce the feed‐forward process has not yet been determined.

The Hippo pathway modulates a variety of biological processes, including survival, differentiation and proliferation, and has been more recently associated with fibrogenesis in pathological wound healing.^10^, ^11^, ^12^ The Hippo pathway contains a kinase cascade that regulates the localization and stability of Yes‐associated protein (YAP) and WW domain‐containing protein (TAZ).^13^ The kinase cascade is activated in the absence of mechanical stress, and YAP/TAZ is rapidly destabilized by proteasome‐induced degradation.^14^ In contrast, the kinase cascade is inactivated in the presence of mechanical stress, and YAP/TAZ is stabilized and translocated to the nucleus.^14^ Following this, YAP/TAZ in the nucleus interacts with the TEA domain (TEAD) transcription factors and promotes the transcription of genes involved in fibrosis, cell survival and cell proliferation. YAP has been reported to act as a fibroblast activator, and researchers have found that YAP mechanically activates the feed‐forward loop in cancer cells.^15^ Furthermore, it has been reported that sustained activation of YAP in renal cells induces ECM production and promotes myofibroblast transformation.^16^ Increased YAP expression was shown to stimulate myofibroblast accumulation and facilitate myofibroblast formation during the development of systemic sclerosis fibrosis.^17^ Therefore, YAP is considered as a determinant of the proliferative capacity of myofibroblasts and promotes their fibrogenetic activity.

In this study, we investigated whether YAP affects the development and progression of TGF‐β‐induced retinal fibrosis in vivo and the activation and proliferation of retinal MCs in vitro. Our findings indicated that YAP activation led to increased ECM deposition, which increased ECM stiffness. Further, our data revealed that the interaction between YAP and ECM forms a feed‐forward loop that leads to retinal fibrosis, and hence, may contribute to identifying optimal targets and developing effective treatments to prevent fibrogenesis in DR.

## 2 MATERIALS AND METHODS

### 2.1 Induction of experimental diabetes

All the experimental protocols of this study were approved by the Medical Ethics Committee of Tianjin Medical University. All animal‐ and cell‐based studies were conducted in accordance with the Declaration of Helsinki and the principles of research of the Association for Research in Vision and Ophthalmology. Experimental diabetes was induced as described previously.^18^ In brief, Wistar rats (8‐week‐old) were injected intraperitoneally with streptozotocin (STZ; 45 mg/kg, Sigma‐Aldrich, St.Louis, MO,USA) after overnight fasting. Serum glucose levels were monitored 48 hours after STZ injection and every 2 weeks thereafter. Only the rats with blood glucose levels >16.7 mmol/L were used as STZ‐induced diabetic rats.

To investigate role of YAP in diabetic rats, 80 rats were divided into the following groups: (a) normal control (Ctrl) group (20 rats); (b) diabetic mellitus (DM)‐week 4 group (20 rats); (c) DM‐week 8 group (20 rats); and (d) DM‐week 16 group (20 rats). To inhibit the expression of YAP in the retina, 60 rats were divided into the following groups: (a) normal control (Ctrl) group (20 rats); (b) DM‐week 16 without verteporfin treatment group (20 rats); and (c) DM‐week 16 treated with verteporfin group (20 rats). We intraperitoneally administered verteporfin (100 mg/kg every other day for 4 weeks) to rats in the DM‐week 16 group. After the rats were killed, the eyes were rapidly enucleated and processed for haematoxylin and eosin staining, immunohistochemistry, and Western blotting as described in the following sections.

### 2.2 Culture and treatment of MCs

The immortalized human MC line (MIO‐M1) was initially derived from the human retina by Dr Limb.^19^ MIO‐M1 cells retain several important characteristics of MCs in situ.^19^ MIO‐M1 cells were cultured in Dulbecco's modified Eagle's medium (Invitrogen, Carlsbad, CA, USA) containing 10% foetal bovine serum (Invitrogen) at 37°C with 5% CO_2_. Cells were then passaged at 80% confluency.

To inhibit the expression of YAP in human retinal Müller cells (hMCs), hMCs were treated with siRNAs against YAP. YAP‐siRNA and non‐silencing siRNA (control‐siRNA, Con‐siRNA) were purchased from Invitrogen. Transient transfection of siRNA was performed with Lipofectamine 2000 transfection reagent (Santa Cruz Biotechnology, USA). To disrupt the YAP‐TEAD interaction, hMCs were treated with verteporfin (Billerica, MA, USA) as per the manufacturer's recommendations. TGF‐β1 (Santa Cruz Biotechnology, Delaware, USA) was used at a dose of 2 ng/mL. The lentiviral particles were packaged and used to infect hMCs. A constitutively active YAP mutant (YAP5SA) was purchased from Invitrogen. YAP5SA has its LATS kinase‐induced phosphorylation sites substituted for alanine to prevent cytoplasmic sequestration and proteasomal degradation.^20^ Next, hMCs were treated with LY294002 (a PI3K inhibitor; Santa Cruz Biotechnology) and MK‐2296 (an Akt inhibitor; Santa Cruz Biotechnology) at a concentration of 10 μM. Cell lysates were prepared 48‐72 hours after lentiviral infection for Western blotting.

### 2.3 Polyacrylamide hydrogels for cell culture

Polyacrylamide hydrogels with high (20‐50 kPa) and low stiffness (0.5‐5 kPa) were used for this study. The production of fibronectin‐coated polyacrylamide hydrogels was performed as described in a previous study.^17^ After hydrogel polymerization, the surface of the gels was coated with collagen I (10 ng/mL; Thermo Fisher Scientific, Thermo Fisher Scientific, Cleveland, OH, USA). The elastic modulus was calculated based on the polyacrylamide concentration.

### 2.4 YAP nuclear localization

YAP localization was detected in hMCs by immunofluorescence analysis as described previously.^21^ Briefly, hMCs were incubated overnight with monoclonal antibodies against YAP (Santa Cruz Biotechnology), followed by incubation with an Alexa Fluor 488‐conjugated secondary antibody (Santa Cruz Biotechnology). Images were obtained using a fluorescence microscope (Olympus Soft Image Solutions GmbH, Hamburg, Germany).

### 2.5 Histological and immunohistochemical measurements

The rat eyes were placed in phosphate‐buffered saline (PBS; pH 7.4) for 2 hours, dehydrated in a graded alcohol system and embedded in paraffin wax. For histopathological analysis, Masson's trichrome staining was performed to test retinal fibrosis following a standard protocol.^22^ Briefly, the slides were placed in a staining jar and deparaffinized by submerging in absolute xylene for 4 minutes. Next, the slides were treated with phosphomolybdic acid solution for 10 minutes as a mordant and immediately submerged into methyl blue (Thermo Fisher Scientific). Following this, the slides were washed in PBS for 1 minute and then treated with 1% acetic acid solution for 2 minutes.

For immunohistochemical analysis, the paraffin‐embedded eye tissues were sliced and incubated with the following antibodies for 2 hours: antibodies against YAP, vimentin, TGF‐β1, fibronectin, collagen I, α‐SMA and connective tissue growth factor (CTGF) (Sigma Chemical Co., USA). Next, the sliced tissues were stained with biotinylated anti‐rat IgG secondary antibodies, followed by incubation with horseradish peroxidase‐conjugated streptavidin for 1 hour. For double immunofluorescence staining, after incubation with primary antibodies, the sliced tissues were incubated with fluorescent secondary antibodies. Images were obtained using the Leica DMI4000B microscope (Leica Biosystems, Vista, CA, USA).

### 2.6 Real‐time PCR

Total RNA was isolated from hMCs using TRIzol reagent and reverse transcribed into cDNA with the SuperScript III First‐Strand Synthesis System (Cambridge, MA, USA). Quantitative PCR was performed with Power SYBR Green PCR Master Mix (Invitrogen). The primer sequences for Real‐time PCR (RT‐PCR) were as follows: β‐actin, forward: 5′‐CGAGAC CACCTTCAACTCGATCAT‐3′, reverse: 5′‐ATCTCCTTCTGCATCCTGTCGG‐3′; YAP, forward: 5′‐CTGGAGGGAGATGGAATGAA‐3′, reverse: 5′‐ATCGCCTTAGCTCCTTCACA‐3′.

### 2.7 Western blotting

Total protein was extracted from the retinal samples and hMCs. As described previously,^20^ nuclear and cytoplasmic extracts were prepared from hMCs using the NE‐PER nuclear and cytoplasmic extraction kit (Santa Cruz Biotechnology). Proteins were quantified with a commercial bicinchoninic acid (BCA) kit. Sodium dodecyl sulphate–polyacrylamide gel electrophoresis was used to separate equal amounts of protein. Antibodies specific for YAP, collagen I, α‐SMA, CTGF, fibronectin, TGF‐β1, p‐Akt, Akt and β‐actin were purchased from Abcam (Cambridge, MA, USA). Target proteins were detected using an enhanced chemiluminescence kit (Santa Cruz Biotechnology). The blots were then imaged using the Olympus Soft Image Solutions software.

### 2.8 Statistical analysis

All data were analysed using spss 16.0 (SPSS Inc, Chicago, IL, USA). Statistical analyses were performed with Student's *t* test for comparisons involving two sets of data and one‐way analysis of variance (ANOVA) or two‐way ANOVA for comparisons involving the means of three groups of data. For each analysis, *P* < 0.05 was considered statistically significant.

## 3 RESULTS

### 3.1 Involvement of YAP in diabetes‐induced retinal fibrogenesis

**Figure 1:**
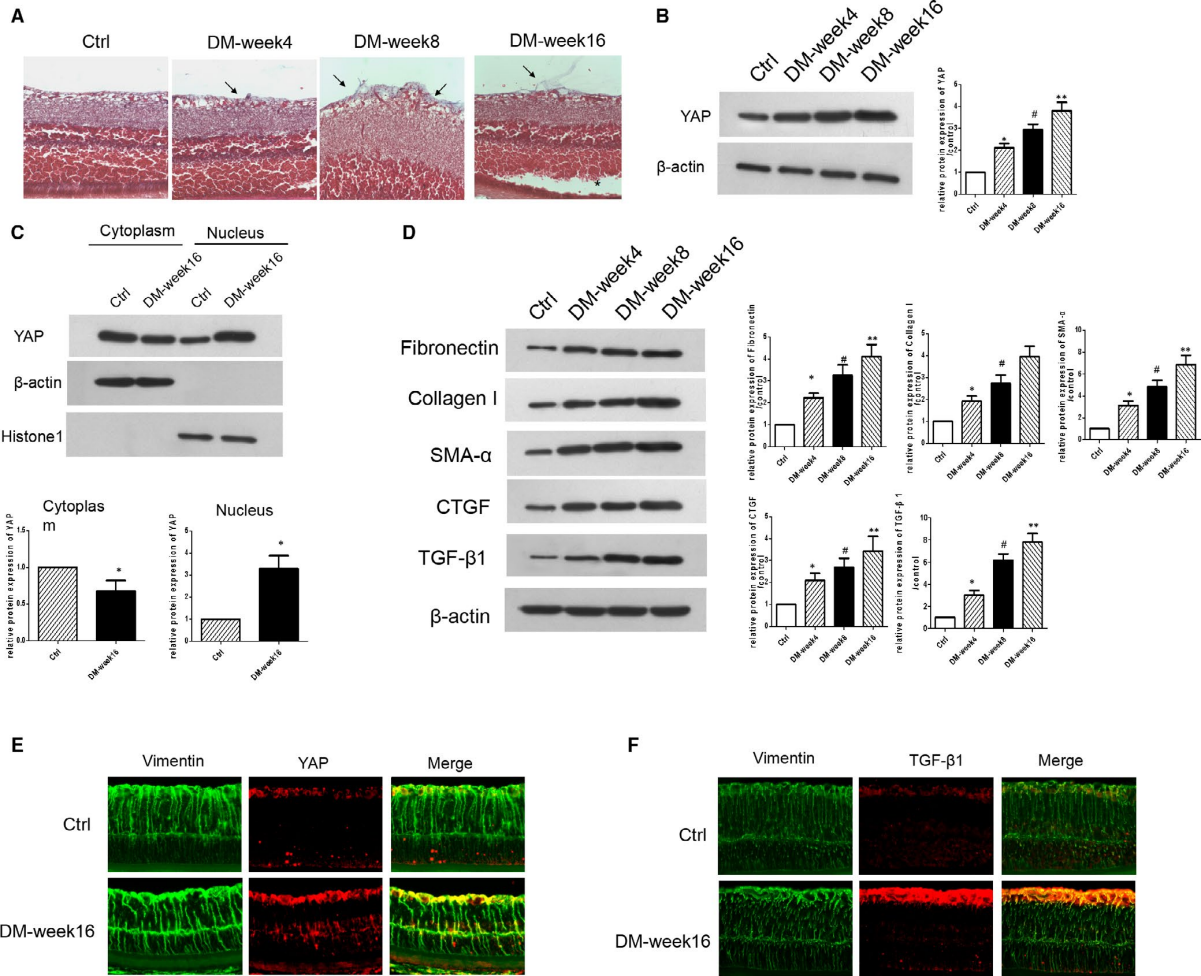
Involvement of YAP in diabetes‐induced retinal fibrogenesis. A, Representative images of Masson's staining in the retinal tissues of different rat groups (the arrow indicates collagen fibres and “*” indicates retinal detachment). B, Western blot analysis for the expression of YAP in the retinal tissues of rats. C, YAP protein expression in the cytoplasmic and nuclear fractions from the rat retinal tissues were detected by Western blotting. D, Western blot analysis for the expression of fibronectin, collagen I, α‐SMA, CTGF and TGF‐β1 in the rat retinal tissues. E, Double immunofluorescence staining of vimentin and YAP in the retinal tissues of different rat groups. F, Double immunofluorescence staining of vimentin and TGF‐β1 in the retinal tissues of different rat groups.*P < 0.05 vs control group; ^#^ *P* < 0.05 vs DM‐week 4 group; **P < 0.05 vs DM‐week 8 group; n = 3 repeats

Masson's staining confirmed the presence of the epiretinal fibrotic membrane (white arrows) and localized retinal detachment (indicated by *) in the DM‐week 8 and DM‐week 16 groups, while collagen fibres (white arrows) were less abundant in the DM‐week 4 and Ctrl groups (Figure 1A). The expression of YAP was greatly increased in the retinal tissues from week 4 to week 16 after DM induction (Figure 1B). Because activated YAP exerts its transcription‐promoting activity in the nucleus, we isolated nuclear and cytoplasmic proteins from the retinal tissues. Results showed that exposure to hyperglycaemia increased YAP expression and most of the YAP protein was located in the nucleus (Figure 1C). Western blot analysis showed increased accumulation of collagen I and fibronectin (ECM markers) and increased expression of CTGF (YAP target) and α‐SMA in the retinal tissues of diabetic rats (Figure 1D). TGF‐β1 is a potent stimulator of myofibroblast activation. Our results also revealed that exposure to hyperglycaemia stimulated the expression of TGF‐β1 in the retinal tissues (Figure 1D). Furthermore, double immunofluorescence staining indicated that exposure to hyperglycaemia increased the accumulation of YAP and TGF‐β1, and most of YAP and TGF‐β1 co‐stained with MCs marker vimentin (Figure 1E,F)

### 3.2 YAP activation induces MC differentiation and ECM production

**Figure 2:**
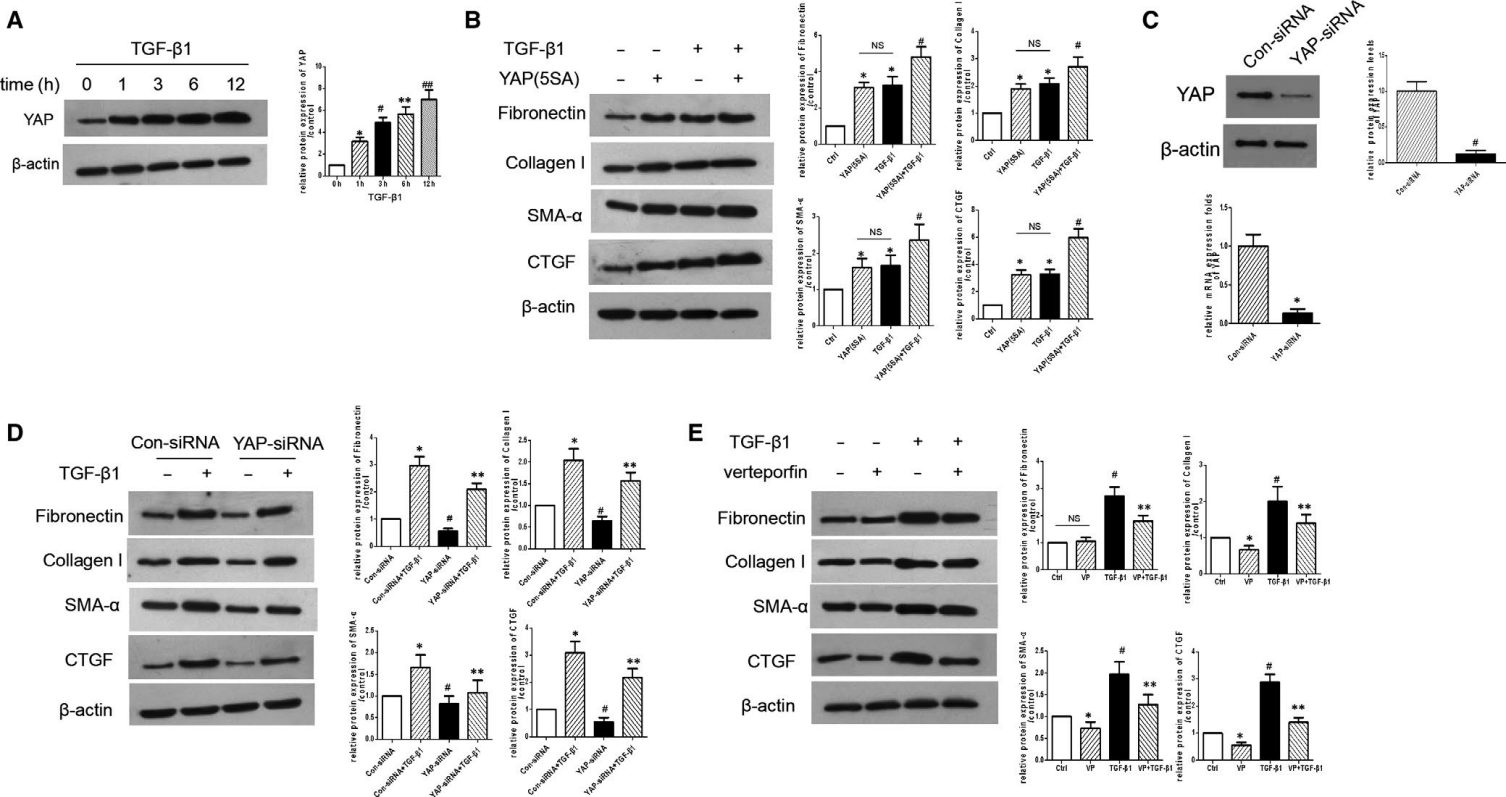
YAP activation induces MC differentiation and ECM production. A, The protein level of YAP in hMCs treated with TGF‐β1 at different time points was detected by Western blotting. ^*^ *P* < 0.05 vs TGF‐β1(0) group; ^#^ *P* < 0.05 vs TGF‐β1(1) group; **P < 0.05 vs TGF‐β1(3) group; ^##^ *P* < 0.05 vs TGF‐β1(6) group. B, The protein levels of fibronectin, collagen I, α‐SMA and CTGF in hMCs expressing constitutive active YAP (YAP5SA) with or without TGF‐β1 treatment were detected by Western blotting. *P < 0.05 vs control group; ^#^ *P *< 0.05 vs TGF‐β1 group. C, YAP expression in hMCs transfected with YAP‐siRNA or Con‐siRNA was detected by RT‐PCR. *P < 0.05 vs Con‐siRNA group. D, The protein levels of fibronectin, collagen I, α‐SMA and CTGF in TGF‐β1‐treated hMCs transfected with YAP‐siRNA or Con‐siRNA were detected by Western blotting. *P < 0.05 vs Con‐siRNA group; ^#^ *P* < 0.05 vs Con‐siRNA + TGF‐β1 group; ^**^ *P* < 0.05 vs YAP‐siRNA group. E, The protein levels of fibronectin, collagen I, α‐SMA and CTGF in TGF‐β1‐treated hMCs with or without vertoporfin treatment were detected by Western blotting. VP = vertoporfin; *P < 0.05 vs control group; ^#^ *P* < 0.05 vs VP group; **P < 0.05 vs TGF‐β1 group; n = 3 repeats

hMCs exhibit the characteristics of fibroblasts, including alterations in cytoskeletal structure and generation of traction force in the retina.^23^ The differentiation of fibroblasts into myofibroblasts is closely associated with the progression of retinal fibrosis. As TGF‐β1 is a well‐known stimulator of myofibroblast activation,^24^ we treated hMCs with TGF‐β1 and studied its effects on the levels of proteins in the Hippo pathway. Our results showed that TGF‐β1 treatment gradually increased the level of YAP protein (Figure 2A). Based on this finding, we further studied the role of YAP in MC activation.

Our results showed that hMCs expressing constitutively active YAP (YAP5SA) promoted fibroblast differentiation and increased the protein levels of fibronectin, collagen I, α‐SMA and CTGF, even in the absence of TGF‐β1, suggesting that YAP activation is essential for the differentiation and activation of fibroblasts and acts independently of TGF‐β1 (Figure 2B). Next, YAP‐siRNA was designed, and its transfection efficiency was verified using RT‐PCR. Transfection with YAP‐specific siRNA dramatically reduced the YAP mRNA levels (~85% knockdown) compared to transfection with Con‐siRNA (Figure 2C). We also examined the protein level of YAP to assess the efficiency of YAP inhibition by YAP‐siRNA in hMCs. YAP expression was markedly decreased in YAP‐siRNA‐treated hMCs (~85% knockdown) compared to that in Con‐siRNA‐treated hMCs. Our results also showed that YAP inhibition in hMCs suppressed TGF‐β1‐induced expression of fibronectin, collagen I, α‐SMA and CTGF (Figure 2D). Furthermore, to investigate the role of YAP signalling in TGF‐β1‐induced fibrogenic response, hMCs were treated with verteporfin, a chemical that can disrupt the YAP‐TEAD interaction.^25^ Results showed that treatment with verteporfin suppressed TGF‐β1‐induced expression of fibronectin, collagen I, α‐SMA and CTGF (Figure 2E). These findings suggest that YAP plays a vital role in the activation and up‐regulation of the fibrogenic activity of MCs.

### 3.3 ECM stiffening stimulates YAP and induces MC activation

**Figure 3:**
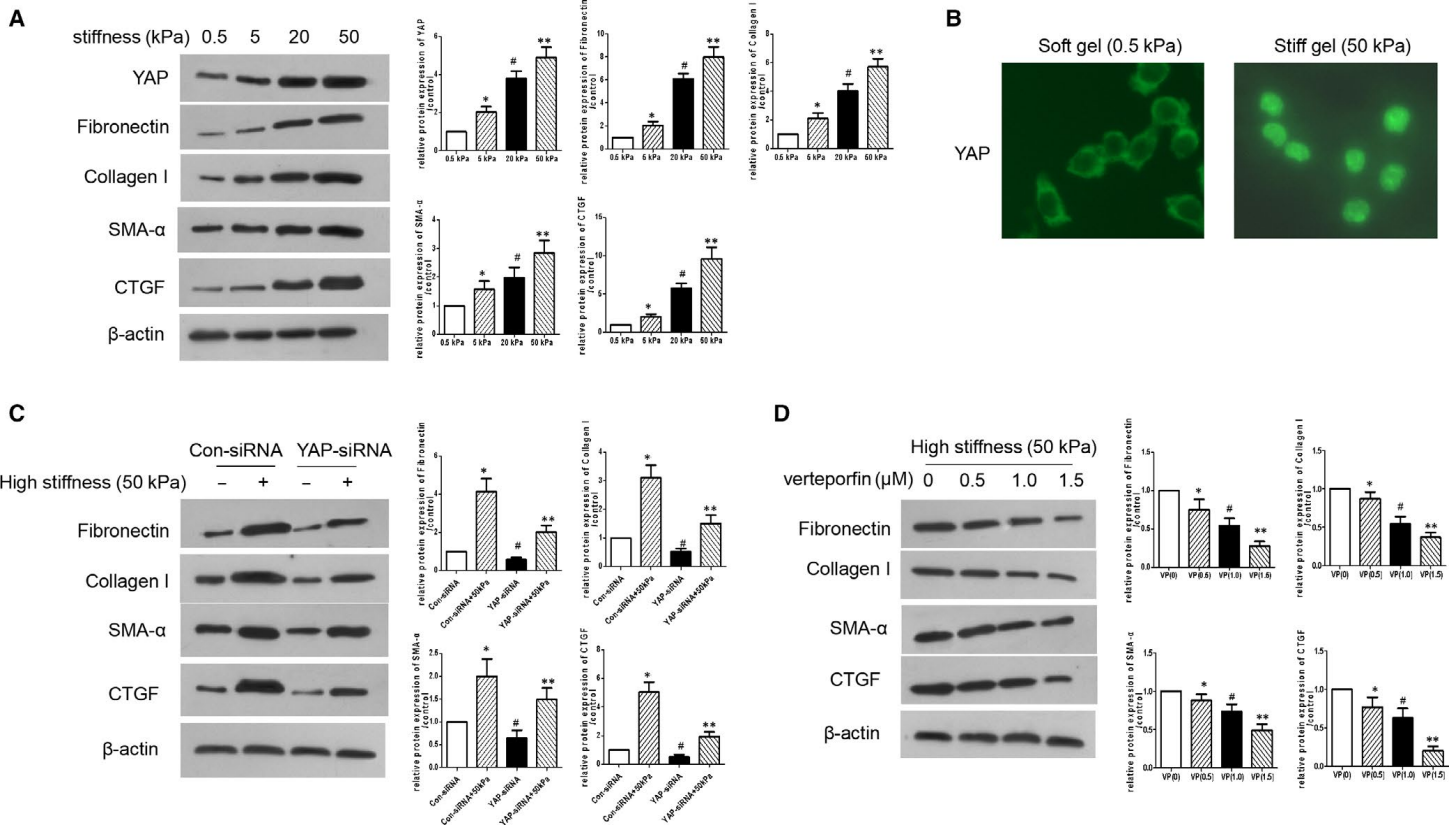
ECM stiffness stimulates YAP and induces MC activation. A, The protein level of YAP, fibronectin, collagen I, α‐SMA and CTGF in hMCs cultured on polyacrylamide gels of varying stiffness was detected by Western blotting. *P < .05 vs 0.5 kPa group; ^#^ *P* < .05 vs 5 kPa group; **P < .05 vs 20 kPa group B, YAP nuclear localization in hMCs cultured on polyacrylamide gels of varying stiffness was determined by immunofluorescence staining. C, The protein levels of fibronectin, collagen I, α‐SMA and CTGF in hMCs transfected with YAP‐siRNA or Con‐siRNA and cultured on polyacrylamide gels of varying stiffness were detected by Western blotting. *P < 0.05 vs Con‐siRNA group; ^#^ *P* < 0.05 vs Con‐siRNA + 50 kPa group; **P < 0.05 vs YAP‐siRNA group. D, The protein levels of fibronectin, collagen I, α‐SMA and CTGF in hMCs cultured on high‐stiffness gels with varied concentrations of verteporfin were detected by Western blotting. VP, vertoporfin; Con‐siRNA, Control‐siRNA (non‐silencing siRNA) *P < 0.05 vs VP(0) group; ^#^ *P* < 0.05 vs VP(0.5) group; ^**^ *P* < 0.05 vs VP(1.0) group; n = 3 repeats

The stiffness of the normal retinal tissue is approximately 4 kPa.^26^ To investigate the effect of ECM stiffness on hMC activation, hMCs were cultured on polyacrylamide gels of varying stiffnesses, but with the top surface conjugated with the same concentration of collagen. This created an environment in which biochemical properties were decoupled from mechanical properties. Our results showed that the protein levels of YAP and its downstream target, CTGF, were increased in an ECM stiffness‐dependent manner (Figure 3A). High matrix stiffness also induced the expression of fibronectin and α‐SMA (Figure 3A). hMCs cultured in gels with a higher stiffness displayed increased YAP nuclear localization, while cells cultured on softer gels showed a decrease in YAP nuclear localization (Figure 3B). In contrast, hMCs transfected with YAP‐siRNA displayed reduced stiffness‐stimulated expression of fibronectin, collagen I, α‐SMA and CTGF (Figure 3C). Furthermore, hMCs cultured on gels with a higher stiffness showed increased expression of fibronectin, CTGF and α‐SMA, as indicated hMCs activation. However, this effect was inhibited by treatment with verteporfin at doses of 0.5‐1.5 mM (Figure 3D). Therefore, our data suggest that matrix stiffening induced hMC activation in a YAP‐dependent manner.

### 3.4 YAP blockade inhibits hyperglycaemia‐induced retinal fibrosis

**Figure 4:**
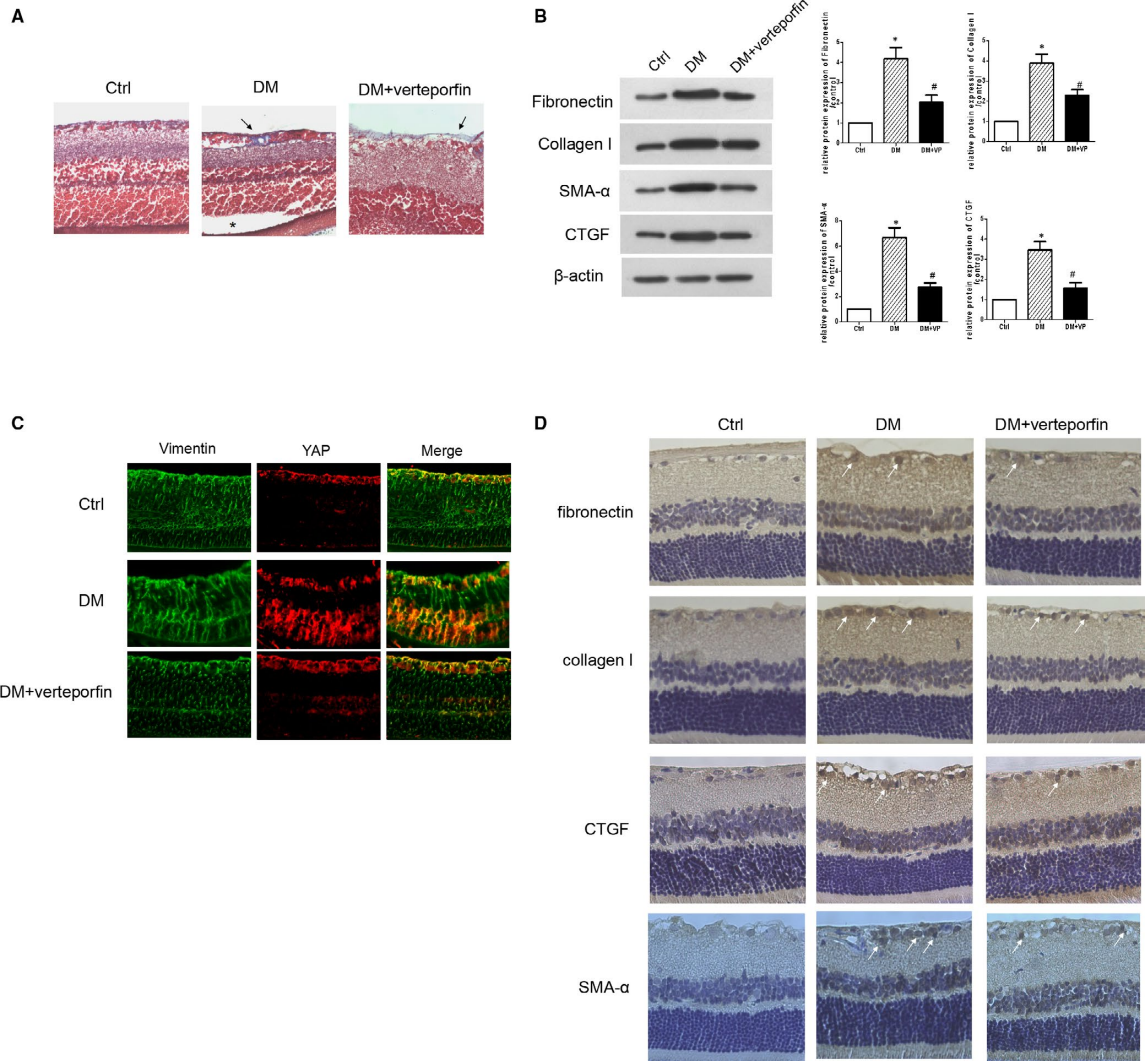
YAP blockade inhibits hyperglycaemia‐induced retinal fibrosis. A, Representative images of Masson's staining in rat retinal tissues with or without vertoporfin treatment (the arrow indicates collagen fibres and “*” indicates retinal detachment) B, Western blot analysis for the expression of fibronectin, collagen I, α‐SMA and CTGF in rat retinal tissues with or without vertoporfin treatment. C, Double immunofluorescence staining of vimentin and YAP in the retinal tissues from different rat groups. D, Representative images of positive staining for fibronectin, collagen I, α‐SMA and CTGF in rat retinal tissues with or without vertoporfin treatment, as examined by immunohistochemistry (arrows indicate positive cells). Quantitative analysis was performed with Image Pro Plus software. VP, vertoporfin; *P < 0.05 vs control group; ^#^ *P* < 0.05 vs DM group; n = 3 repeats

To investigate whether YAP blockade ameliorates hMC activation in vivo, rats in the DM‐week 16 group were intraperitoneally administered verteporfin (100 mg/kg every other day for 4 weeks). Masson's trichome staining confirmed the presence of the epiretinal fibrotic membrane (white arrows) and localized retinal detachment (indicated by *) in the DM‐week 16 group. However, no apparent retinal detachment and reduced formation of the epiretinal fibrotic membrane (white arrows) were observed in the verteporfin treatment group (Figure 4A). In addition, the expression of fibronectin, collagen I, α‐SMA and CTGF was partially inhibited by treatment with verteporfin (Figure 4B). Double immunofluorescence staining indicated that verteporfin treatment decreased the accumulation of YAP in diabetic rats (Figure 4C), and most of YAP co‐stained with MCs marker vimentin. While immunohistochemical analysis showed that verteporfin treatment reduced the levels of fibronectin, collagen I, α‐SMA and CTGF (Figure 4D).

### 3.5 PI3K/Akt pathway mediates YAP activation in MCs

**Figure 5:**
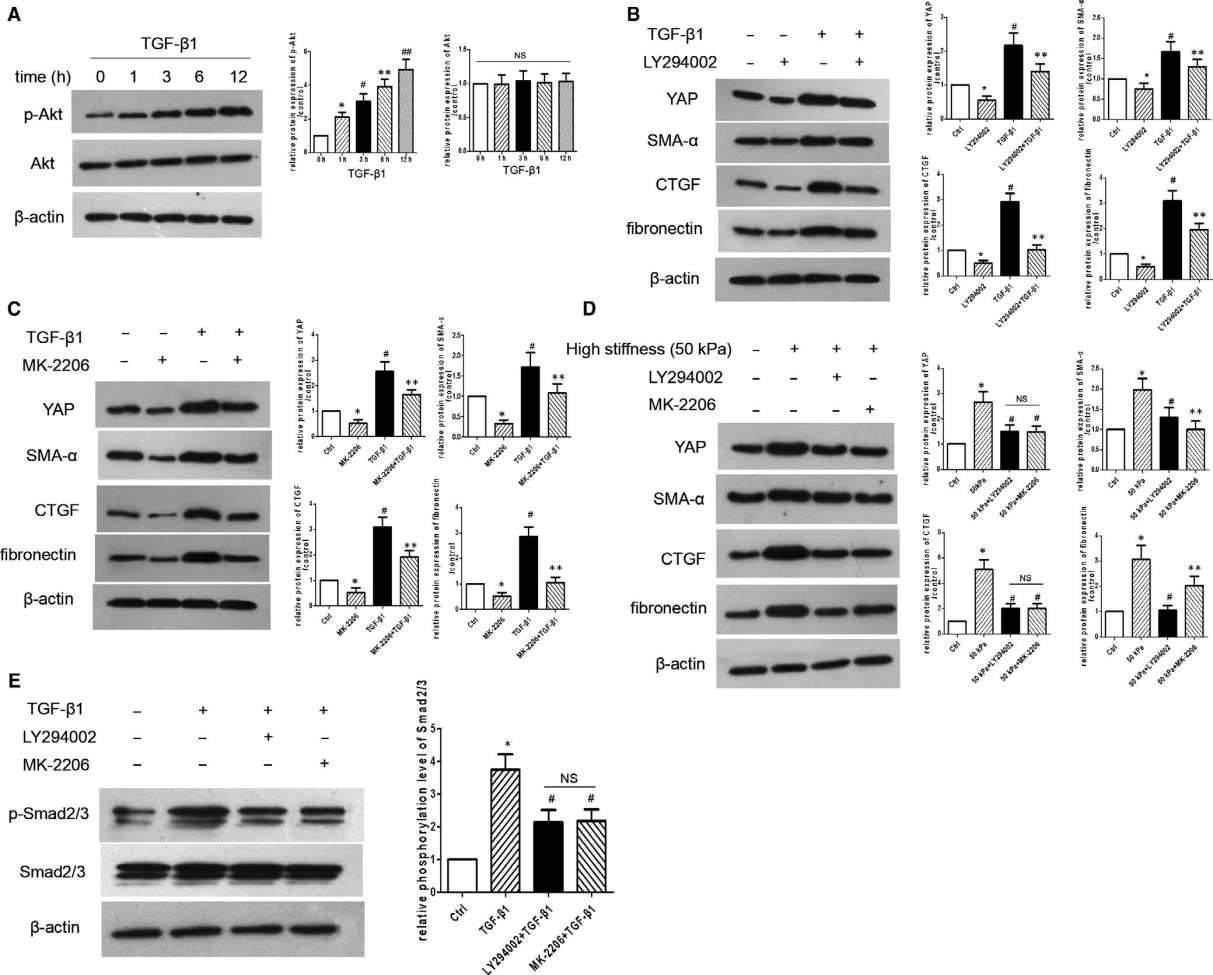
The PI3K/Akt pathway mediates YAP activation in MCs. A, The protein level of p‐Akt and Akt in hMCs treated with TGF‐β1 at different time points was detected by Western blotting. *P < 0.05 vs TGF‐β1(0) group; ^#^ *P* < 0.05 vs TGF‐β1(1) group; **P < 0.05 vs TGF‐β1(3) group; ^##^ *P* < 0.05 vs TGF‐β1(6) group. B, The protein levels of YAP, fibronectin, collagen I, α‐SMA and CTGF in hMCs pre‐treated with or without LY294002 and then exposed to TGF‐β1 were detected by Western blotting. *P < 0.05 vs control group; ^#^ *P* < 0.05 vs LY294002 group; **P < 0.05 vs TGF‐β1 group. C, The protein levels of YAP, fibronectin, collagen I, α‐SMA and CTGF in hMCs pre‐treated with or without MK‐2206 and then exposed to TGF‐β1 were detected by Western blotting. *P < 0.05 vs control group; ^#^ *P* < 0.05 vs MK‐2206 group; **P < 0.05 vs TGF‐β1 group. D, The protein levels of fibronectin, collagen I, α‐SMA and CTGF in hMCs pre‐treated with or without MK‐2206/LY294002 and then cultured on high‐stiffness gels were detected by Western blotting. *P < 0.05 vs control group; ^#^ *P* < 0.05 vs 50 kPa group; **P < 0.05 vs 50 kPa + LY294002 group. E, The protein levels of p‐Smad2/3 and Smad2/3 in hMCs pre‐treated with or without MK‐2206/LY294002 and then exposed to TGF‐β1 were detected by Western blotting. *P <0 .05 vs control group; ^#^ *P* < 0.05 vs TGF‐β1 group; n = 3 repeats

A recent study indicated that the PI3K/Akt pathway may be involved in promoting the nuclear localization of YAP in epithelial cells.^27^ Thus, we examined the underlying mechanism of YAP expression in response to TGF‐β1. As shown in Figure 5A, treatment with TGF‐β1 resulted in a significant increase in Akt phosphorylation. In contrast, inhibition of PI3K with LY294002 not only blunted the expression of YAP, but also inhibited TGF‐β1‐induced expression of α‐SMA, CTGF and fibronectin (Figure 5B). To evaluate the role of Akt in YAP activation in TGF‐β1‐treated MCs, cells were treated with the Akt inhibitor MK‐2206, which resulted in reduced YAP expression and subsequent decrease in TGF‐β1‐induced CTGF and α‐SMA expression (Figure 5C). We further investigated whether the PI3K/Akt pathway is involved in high ECM stiffness‐induced hMC activation. As shown in Figure 5D, treatment with LY29002 and MK‐2206 significantly reduced matrix stiffness‐induced expression of fibronectin, collagen I, CTGF and α‐SMA. Next, to explore the role of TGF‐β1/Smad2/3 signalling, we performed Western blotting for p‐Smad2/3 and Smad2/3. Our results showed that treatment with TGF‐β1 increased the phosphorylation of Smad2/3, whereas treatment with LY29002 and MK‐2206 significantly decreased TGF‐β1‐induced increase in Smad2/3 phosphorylation (Figure 5E). These data indicate that YAP expression is mediated by a PI3K/Akt‐dependent pathway in MCs.

## 4 DISCUSSION

**Figure 6:**
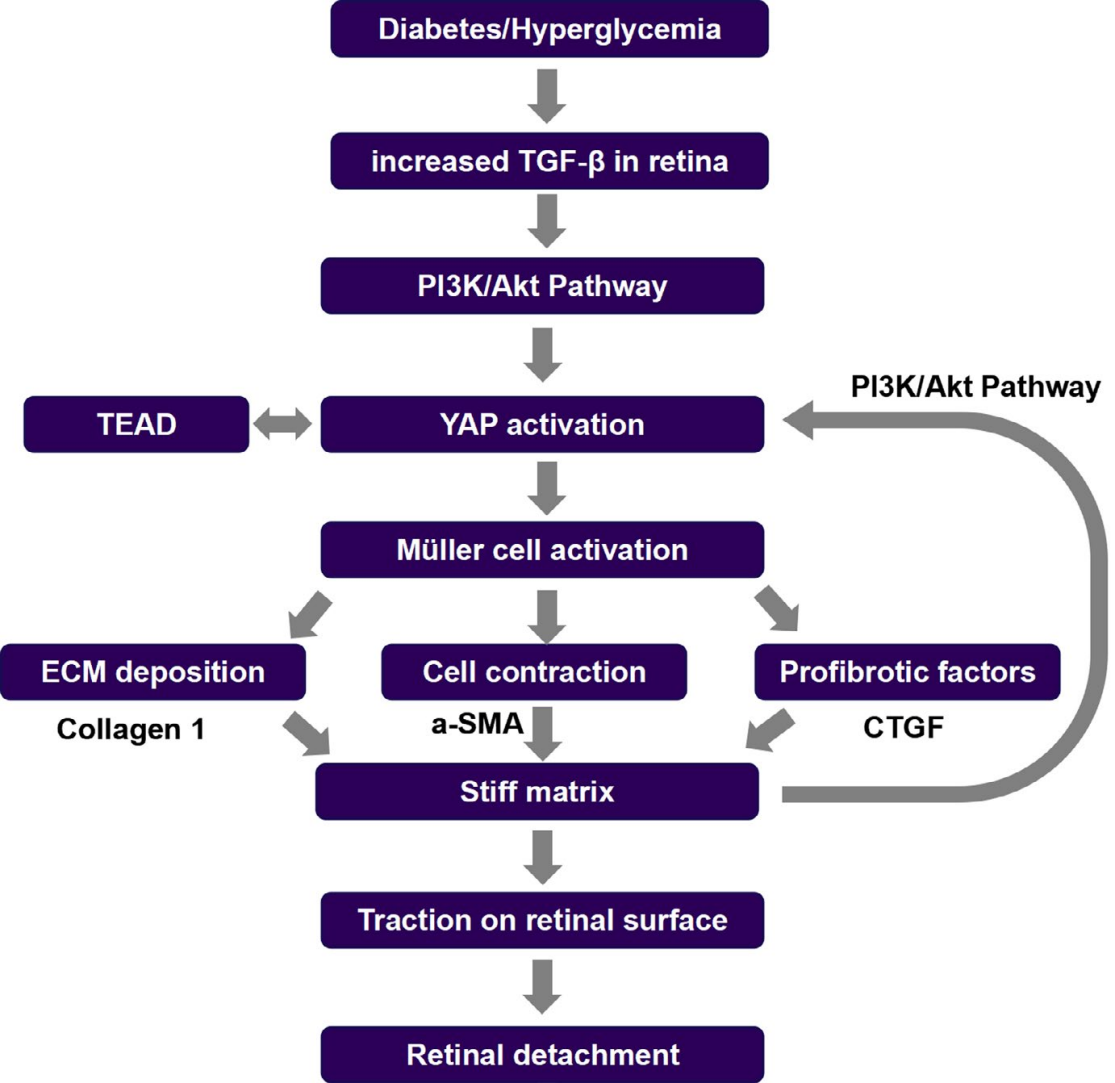
The interaction between ECM stiffness and YAP could form a feed‐forward process, leading to retinal fibrosis in DM. Under hyperglycaemic conditions, TGF‐β1 expression increases in the retina, which leads to activation of PI3K/Akt signalling. Next, YAP is activated and translocated to the nucleus, where it interacts with the TEAD family of transcription factors. In MCs, YAP acts as a sensor of ECM stiffness via a mechanical transduction pathway. YAP also stimulates the generation of fibrogenic factors and ECM proteins, thus enhancing cell contraction. This response enhances matrix stiffness in the retina and forms a feed‐forward loop for MC activation and retinal fibrosis

In our study, we demonstrated that YAP is crucial for hyperglycaemia‐induced retinal fibrosis via promoting the fibrogenic activity of MCs in vivo and in vitro. In TGF‐β1‐stimulated hMCs, we found that YAP activation was induced in a matrix stiffness‐dependent manner. Our results also indicated that YAP initiates the differentiation of hMCs into myofibroblasts and increases ECM stiffness to maintain its activated state. This creates a feed‐forward loop that leads to progression of retinal fibrosis. However, the forward cycle can be suppressed by inhibiting YAP expression in hMCs by YAP‐siRNA. Next, we demonstrated that treatment with verteporfin, a YAP inhibitor, suppresses the progression of retinal fibrosis induced by hyperglycaemia. As shown in Figure 6, under hyperglycaemic conditions, TGF‐β expression increased in the retinal tissues, leading to the activation of PI3K/Akt signalling. YAP is then activated and translocated to the nucleus, where it interacts with the TEAD family of transcription factors. In MCs, YAP acts as a sensor of ECM stiffness via a mechanical transduction pathway. YAP also stimulates the generation of fibrogenic factors and ECM proteins, thus enhancing cell contraction. This response enhances matrix stiffness in the retina and forms a feed‐forward loop for MC activation and retinal fibrosis.

The origin of myofibroblasts, which cause excessive and uncontrolled generation of ECM proteins in the epiretinal membrane in PDR, remains unknown. Studies on several fibrotic diseases have shown that activated myofibroblasts or fibroblasts have different origins depending on the pathologic process.^28^, ^29^ hMCs have been reported to function as fibroblasts in the retina.^7^ In the normal retinal tissue, hMCs are quiescent and localized to the inner layer of the retina. However, in DR, hMCs are exposed to several growth factors (such as PDGF and TGF‐β), which are derived from injured endothelial cells or plasma platelets and induce ECM deposition.^9^ After activation, hMCs express various markers of myofibroblasts (such as collagen I and α‐SMA), which is consistent with our results. Inhibition of hMC activation has been shown to ameliorate fibrosis in vitro and rescue retinal fibrosis in vivo.^30^ Here, we found that inhibition of the YAP signalling pathway greatly suppressed hMC activation and blocked TGF‐β‐induced fibrosis.

It has been reported that the TGF‐β1/Smad pathway can induce cardiac fibroblast‐to‐myofibroblast transformation.^31^ Here, after YAP inhibition in hMCs, suppression of the TGF‐β1 pathway and myofibroblast activation were observed. YAP has been shown to be involved in the regulation of the TGF‐β1 signalling pathway, potentially through directly binding Smad2/3 to enhance renal fibroblast activation.^32^ Moreover, overexpression of YAP has been demonstrated in lung, renal and liver fibrosis.^33^, ^34^, ^35^ It is worth noting that YAP has been linked to the TGF‐β1/Smad pathway in mouse models of kidney fibrosis.^36^ Consistent with previous studies, our findings also indicated that YAP plays an important role in TGF‐β1‐mediated retinal fibrosis. TGF‐β1 treatment greatly increased YAP protein levels, while pharmacological and genetic inhibition of YAP suppressed TGF‐β1‐stimulated ECM production. This suggests that YAP activation causes ECM production, which in turn maintains the activated state of YAP and creates a feed‐forward loop for retinal fibrosis.

Matrix stiffening was shown to stimulate YAP activation through a Rho/ROCK dependent mechanism in ischaemic heart disease, leading to YAP nuclear accumulation.^37^ Other contractility‐promoting stimuli, such as TGF‐β1 and lysophosphatidic acid have been shown to increase the nuclear localization of YAP in human renal proximal tubular epithelial HK2 cells.^34^ Increased YAP can not only sense ECM deposition and stiffness, but also induce the production of CTGF, which is a downstream target of YAP‐TEAD and a strong pro‐fibrotic factor that induces fibroblast activation and ECM deposition. This response forms a forward loop to stimulate the proliferation and differentiation of hMCs. Moreover, ECM stiffening was shown to stimulate YAP nuclear accumulation and promote skin fibroblast differentiation in systemic sclerosis.^17^ Therefore, our findings suggest that YAP has emerged as a mechanical transducer that transforms mechanical signals into biochemical signals and promotes the differentiation and survival of hMCs.

Retinal fibrosis is suppressed when YAP expression is blocked. In addition, a decrease in the matrix stiffness results in myofibroblast inactivation and quiescence in kidney fibrosis, suggesting that the modulus of matrix stiffness alone can affect the fate of fibroblasts.^36^ In our study, activation of YAP induced hMC proliferation and promoted the progression of retinal fibrosis. Verteporfin is a YAP/TEAD inhibitor that can reduce the abundance of YAP/TEAD.^38^ Here, treatment with verteporfin successfully blocked the feedback loop and suppressed excessive ECM production via hMC activation. Our results also showed that TGF‐β‐induced fibrosis can be reversed by inhibiting YAP signalling, suggesting that suppression of YAP can inhibit or reverse retinal fibrosis. Simultaneously, we also detected the protein levels of the main fibrogenesis‐related signalling molecules. The results showed that Akt was phosphorylated to activate the fibrogenesis signalling pathway. Furthermore, down‐regulation of Akt expression and pharmacologic inhibition of PI3K/Akt activation inhibited TGF‐β1‐dependent YAP expression. This suggests that fibrosis is mediated through the PI3K/Akt signalling pathway via activation of YAP and increased expression of CTGF and fibronectin.

In summary, this study showed that YAP plays an important role in TGF‐β1‐induced retinal fibrosis, and increased matrix stiffness can stimulate YAP activation and induce sustained hMC activation and differentiation into myofibroblasts. Further, our results showed that ECM production promotes myofibroblast proliferation, which in turn increases ECM stiffness and stimulates YAP activation. This feed‐forward process results in retinal fibrogenesis. Hence, YAP inhibition may be a potential strategy for inhibiting fibrosis. Further studies characterizing the function of YAP in vivo are needed in the future.

## CONFLICT OF INTEREST

None.

## AUTHOR CONTRIBUTIONS

**Wei Zhang**: Formal analysis (equal); Validation (equal); Visualization (equal); Writing‐original draft (equal); Writing‐review & editing (equal). **Yichun Kong**: Conceptualization (equal); Data curation (equal); Formal analysis (equal); Funding acquisition (equal); Methodology (equal); Software (equal); Supervision (equal); Validation (equal); Writing‐review & editing (equal).

## Supporting information

Fig S1
