# Supplementary figures and images for: YAP is essential for TGF‐β‐induced retinal fibrosis in diabetic rats via promoting the fibrogenic activity of Müller cells
Source: J Cell Mol Med. 2020 Sep 20;24(21):12390–400. doi: 10.1111/jcmm.15739 (PMC7686973; doi:10.1111/jcmm.15739)

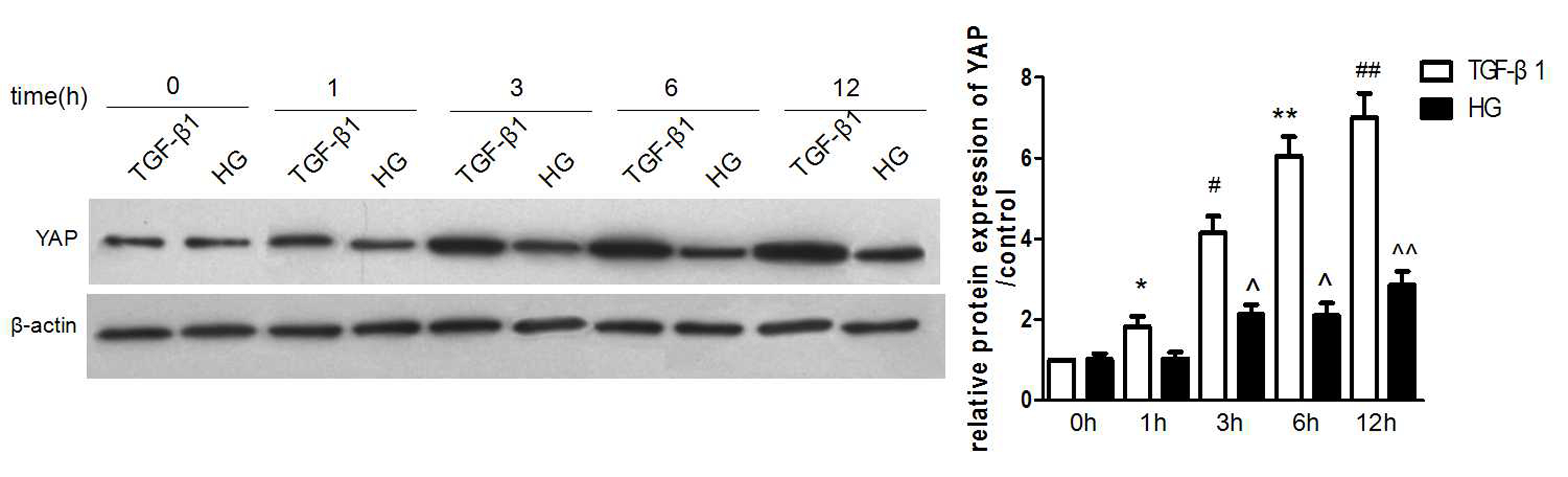

Supplement: Supplementary file 1 — Fig S1 [file JCMM-24-12390-s001.jpg]
